# Does Simultaneous Administration of Bivalent (Types 1 and 3) Oral Poliovirus Vaccine and Inactivated Poliovirus Vaccine Induce Mucosal Cross-immunity to Poliovirus Type 2?

**DOI:** 10.1093/cid/ciy604

**Published:** 2018-10-30

**Authors:** Sonia Resik, Alina Tejeda, Ondrej Mach, Magile Fonseca, Manuel Diaz, Nilda Alemany, Lai Heng Hung, Yoan Aleman, Ileana Mesa, Gloria Garcia, Roland W Sutter

**Affiliations:** 1 Pedro Kouri Institute of Tropical Medicine, Havana, Cuba; 2 Provincial Center of Hygiene, Epidemiology and Microbiology, Camaguey, Cuba; 3 The World Health Organization, Geneva, Switzerland

**Keywords:** poliomyelitis, Cuba, mucosal immunity, immunogenicity, seroconversion

## Abstract

**Background:**

Inactivated poliovirus vaccine (IPV) alone does not induce mucosal immunity. However, it was hypothesized that administration of IPV together with bivalent (types 1+3) oral poliovirus vaccine (bOPV) may stimulate mucosal cross-immunity to poliovirus type 2 (PV2).

**Methods:**

Cuban infants were randomized to receive either one dose of IPV (Arm A); one dose of IPV with bOPV (Arm B) at about 6 months of age or no vaccine (Arm C). Subjects were challenged with one dose of trivalent OPV (tOPV); they were about 7 months old in arms A and B, and about 3 months old in arm C at a time of the tOPV challenge. Sera were collected before vaccination and 30 days after tOPV challenge and tested for presence of poliovirus neutralizing antibodies; stool samples were collected at days 0, 7, 14, 21 and 49 post-challenge and tested for presence of poliovirus.

**Results:**

We enrolled 333 children. Excretion of PV2 following tOPV challenge was highest on day 7 (75 [CI 95% = 65-82%], 68 [CI 95% = 58-75%] and 73 [CI 95% = 63-80%] for study arms A, B, and C respectively); excretion decreased with every subsequent stool sampling; no significant differences either in proportion of PV2 excretion or in its duration were observed between study arms.

**Conclusions:**

There was no reduction in excretion of PV2 after tOPV challenge in children who had received IPV with bOPV when compared to those who had received IPV alone or no vaccine. Polio eradication program cannot assume any PV2 mucosal response with the current polio immunization schedule.

**Clinical Trials Registration:**

The trial was registered with the Australian New Zealand Clinical Trials Registry and allocated trial number ACTRN12616000169448.

The global program to eradicate poliovirus achieved remarkable success in 2017: only 22 children residing in the remaining endemic countries (Pakistan and Afghanistan) were reported to have been paralyzed with the wild poliovirus type 1 [1]. In addition, indigenous wild poliovirus type 2 was last detected in Northern India in 1999 and was declared eradicated in 2015 [2], and wild type 3 poliovirus was last reported from Nigeria in November 2012 and is also likely eradicated [3].

In order to achieve complete poliovirus eradication, it is necessary to develop strategies for the elimination of all polioviruses, including the attenuated Sabin vaccine viruses emanating from oral poliovirus vaccines (OPVs). This is because Sabin-derived polioviruses contained in OPVs can replicate for prolonged periods in individuals or in communities, and potentially re-establish endemic and epidemic transmission, becoming circulating vaccine-derived polioviruses (cVDPVs) [4, 5].

Continued use of OPVs is therefore incompatible with polio eradication and OPVs need to be withdrawn from use in all countries. Phased withdrawal of Sabin strains from OPVs has begun, starting with type 2. This involved a globally-synchronized switch from trivalent OPV (tOPV) to bivalent OPV (bOPV; which includes only Sabin types 1 and 3), which took place in April and May 2016 [6]. In addition to bOPV immunizations, at least 1 full or 2 fractional doses of inactivated poliovirus vaccine (IPV) were recommended to be introduced in routine immunization programs of all countries in order to provide an immunity base to poliovirus type 2 [7]. Unlike OPV, however, IPV does not induce mucosal immunity in poliovirus-naive children [8, 9] and, therefore, it was assumed that children vaccinated with a combination of bOPV and IPV will have no intestinal immunity against poliovirus type 2 (PV2). In a population with low or non-existent intestinal immunity to PV2, the poliovirus (Sabin 2, cVDPV2, or wild type 2) could re-establish silent (or symptomatic) transmission [10, 11].

In previous studies, it was observed that children vaccinated with bOPV and IPV or with bOPV alone demonstrated some degree of mucosal and humoral immunity to type 2; however, it was not clear whether this effect was a result of the cross-reactivity of bOPV and PV2, whether bOPV’s contact with the mucosal surface was able to enhance IPV’s mucosal response, even to PV2 [12, 13], or whether this effect was due to secondary environmental exposure to PV2 among the children enrolled in these studies [14, 15].

In our study, we evaluated whether bOPV could induce a cross-mucosal immunity to PV2 when administered together with IPV, by comparing the mucosal response after IPV was administered either alone or together with bOPV. In addition, we assessed humoral immune responses to bOPV, tOPV, and IPV in Cuban children.

The study was carried out in Camaguey, Cuba, between October 2015 and April 2016. Cuba provided a unique setting in which to evaluate both the serological and mucosal immunity because of the absence of wild polioviruses since 1962 and its unique strategy for administering OPVs in annual campaigns. OPVs are administered to children through biannual National Immunization Drives (NIDs). Each NID round lasts approximately 1 week during January (or February) and March (or April), targeting all children aged greater than 1 month and less than 3 years. The NIDs in 2016 consisted of 2 rounds: the first was conducted 1–7 February 2016 and the second 28 March– 3 April 2016. This was the last use of tOPV in Cuba prior to the global switch to bOPV.

OPVs in Cuba are not available at any other time of the year than during the NIDs. Previous studies in Cuba have shown that any vaccine virus disappears rapidly from the population subsequent to the second round of NID [16]. For these reasons, the potential for “contamination” of the study arms by circulating OPV-derived strains is low, if not entirely eliminated.

In 2015, 1 dose of IPV was introduced into the Cuban routine immunization schedule and was administered at 4 months of age; therefore, some of the children received bOPV before IPV and others received IPV before bOPV, depending on when they were born in relation to the national campaigns with bOPV.

## METHODS

This was a randomized, controlled trial in which children born between 1 June 2015 and 31 August 2015 in the catchment area of the Camaguey province were randomized into 1 of 2 study arms. In arm A, children were assigned to receive IPV as part of their routine immunization at the age of 4 months; in arm B, children were assigned to receive IPV together with 1 dose of bOPV at the age of 4 months. However, due to an IPV vaccine shortage, most of the children in our study received these vaccines at the age of 6 months instead of 4 months. In addition to study arms A and B, we included study arm C, which was comprised of children born between 1 November and 31 December 2015; these children had not received any poliovirus vaccines prior to the NIDs in 2016.

Children in all arms received 2 doses of tOPV as part of the NIDs in 2016; 1 dose each in February and in March/April 2016. The first tOPV dose served as a challenge. Peripheral blood was collected at enrollment and prior to the first and second tOPV doses (for children in arm C, enrollment coincided with the first tOPV dose: therefore, children in arm C provided only 2 blood samples). Stool samples were collected on the day of the first tOPV dose (day 0); 7, 14, and 21 days after the first dose; and on the day of the second tOPV dose (which was 49 days after the first tOPV dose). Each child therefore provided 5 stool samples.

The selection of children was performed using available lists in participating health centres in the Camaguey Province. All children within the desired age groups were screened for eligibility in the study; the parents of those found eligible were asked for consent and, if the parents consented, the children were enrolled. Exclusion criteria were <10 percentile for height and weight; fever or any infectious disease at the time of vaccination; congenital defects; families expecting to move away during the study period; or a diagnosis, suspicion, or treatment of an immunodeficiency disorder (either in the participant or in a member of the immediate family).

Blood samples were collected from subjects using a heel-stick device and were then centrifuged. Sera were transported to Camaguey central laboratory for storage at -20°C until shipment to the Pedro Kouri Institute, Havana. Sera were tested at the Pedro Kouri Institute for neutralizing antibodies against all 3 poliovirus types, using standard neutralization assays [17]. For each serotype, seropositivity was defined as the reciprocal titer of poliovirus neutralizing antibodies ≥8. Seroconversion in children with no maternal antibodies was defined as the change from seronegative to seropositive (from reciprocal titer of <8 to ≥8). In those with maternal antibodies, it was defined as a 4-fold rise in reciprocal titers over the expected decline in maternal antibodies, with an estimated half-life of 28 days. Stool specimens were tested for the presence of poliovirus using real-time polymerase chain reaction (PCR) methodology [18, 19]. Mucosal immunity was defined as a resistance to the excretion of poliovirus after the tOPV challenge [20]. Duration of excretion was defined as the length of uninterrupted excretion of a type-specific poliovirus in the same individual.

We compared the different study outcomes using the Chi-squared testand the distributions of titers using an analysis of variance (ANOVA) test. *P* values ≤ 0.05 were considered significant. All analyses were conducted using the statistical application EpiInfo 7.

## RESULTS

We approached the parents of 352 children and enrolled 333/352 (95%) children, with 113, 116, and 104 in study arms A, B, and C, respectively. All of the children provided blood samples and 330/333 (99%), 324/333 (97%), 325/333 (98%), 323/333 (97%), and 316/333 (95%) provided stool samples on the day of the first tOPV vaccination and 7, 14, 21, and 49 days later, respectively.

Basic demographic data are shown in Table 1. At enrollment, the median age of the children in arms A and B was 6.2 months and the median age in Arm C was 2.5 months. Baseline seroprevalence of maternal antibodies was <10% for all serotypes in study arms A and B. There was no statistical difference in the baseline seroprevalence of maternal antibodies between arms A and B. In arm C, the baseline maternal antibody seroprevalence was between 10–40%. Final seroprevalence included vaccination with 1 dose of IPV and tOPV in arm A; 1 dose of IPV, bOPV, and tOPV in arm B; and 1 dose of tOPV in arm C. The final seroprevalence ranged between 94–97%, 91–96%, and 91–96% for serotypes 1, 2, and 3, respectively (Table 1). There were no significant differences in the final seroprevalence between the study arms; however, the median titer for PV1 was significantly higher in arm B than in the other 2 arms (ANOVA *P* <0.001).

**Table 1.** Basic Demographic Indicators and Baseline and Final Seroprevalence of Anti-polio Antibodies, Including Median Titer and 95% CI

	Arm A (IPV Only)	Arm B (IPV + bOPV)	Arm C (No Vaccine Prior to tOPV)
Demographic indicators			
No. female subjects/total subjects (%)	47/113 (42%)	65/116 (56%)	51/104 (49%)
Age at Enrollment, in months (median IQR)	6.2 (5.6–6.7)	6.2 (5.4–7)	2.5 (2.2–2.8)
Weight, in kgs (median IQR)	8.2 (7.7–8.9)	7.9 (7.3–8.8)	5.7 (5.2–6.5)
Baseline seroprevalence			
Poliovirus Type 1, n/N (%, 95% CI)	8/113 (7%, 3–13%)	3/116 (3%, 0–7%)	41/104 (39%, 30–49%)
Titer, as median (95% CI)	<8 (<8–<8)	<8 (<8–<8)	<8 (<8–<8)
Poliovirus Type 2, n/N (%, 95% CI)	6/113 (5%, 2–11%)	4/116 (3%, 1–9%)	37/104 (36%, 26–46%)
Titer (median, 95% CI)	<8 (<8–<8)	<8 (<8–<8)	<8 (<8–<8)
Poliovirus Type 3, n/N (%, 95% CI)	6/113 (5%, 2–11%)	1/115 (1%, 0–5%)	10/103 (10%, 5–17%)
Titer, as median (95% CI)	<8 (<8–<8)	<8 (<8–<8)	<8 (<8–<8)
Final seroprevalence			
Poliovirus Type 1, n/N (%, 95% CI)	109/113 (96%, 91–99%)	109/116 (94%, 88–98%)	101/104 (97%, 92–99%)
Titer, as median (95% CI)	283, 179–508	897, 713–1130	449, 283–566
Poliovirus Type 2, n/N (%, 95% CI)	109/113 (96%, 91–99%)	105/116 (91%, 84–95%)	99/104 (95%, 89–98%)
Titer (median, 95% CI)	357, 225–449	320, 225–357	225, 179–283
Poliovirus Type 3, n/N (%, 95% CI)	108/112 (96%, 91–99%)	105/116 (91%, 84–95%)	100/104 (96%, 90–99%)
Titer, as median (95% CI)	805 (566–1130)	449 (357–566)	283 (142–449)

Abbreviations: bOPV, bivalent oral poliovirus vaccine; CI, confidence interval; IPV, inactivated poliovirus vaccine; IQR, interquartile range; tOPV, trivalent oral poliovirus vaccine.

**Figure 1.**
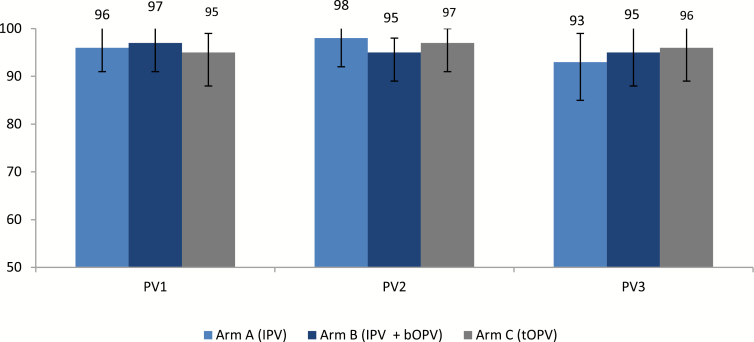
Seroconversion (% and 95% confidence interval]) after 1 dose of IPV (Arm A), 1 dose of IPV+bOPV (Arm B), or 1 dose of tOPV (Arm C). Abbreviations: bOPV, bivalent oral poliovirus vaccine; IPV, inactivated poliovirus vaccine; PV1–3, polyvirus types 1–3; tOPV, trivalent oral poliovirus vaccine.

We measured seroconversion in study arms A (after 1 dose of IPV) and B (after 1 dose of IPV and bOPV; Figure 1). There was no statistical difference in the proportion of children who seroconverted between study arms A and B. However, there was a statistical difference in the median reciprocal antibody titer for serotype 1: it was 17 and 449 for study arms A and B, respectively (ANOVA *P* <0.001). There was no significant difference for serotype 3 (titer: 44 vs 71; ANOVA *P* = .25).

Seroconversion after 1 dose of tOPV (in study arm C) was 95, 97, and 96% for serotypes 1, 2, and 3, respectively (Figure 1). For serotypes 1 and 3, seroconversion was significantly lower among those with detectable maternal antibodies than among those who did not have maternal antibodies at baseline (PV1: 83% vs 97%; PV3: 64% vs 96%; *P* <0.01); however, this significance was absent for serotype 2 (PV2: 95% vs 94%; *P* = .8).

**Figure 2.**
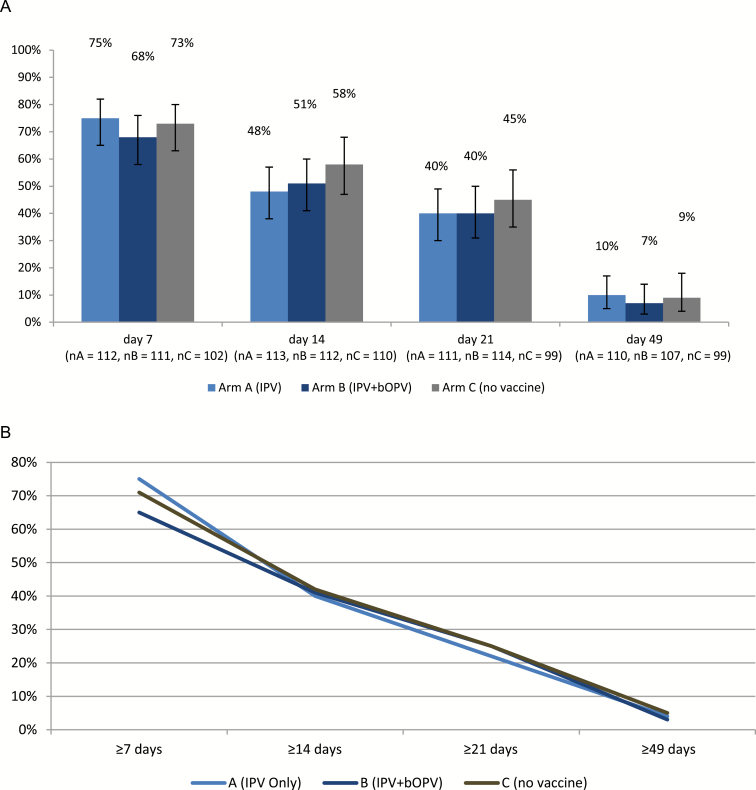
Excretion of PV2 following challenge with tOPV. *A*, Proportion of children shedding PV2 at each visit post–tOPV challenge. *B*, Duration of excretion of PV2 post–tOPV challenge (expressed as % children with uninterrupted excretion of type specific poliovirus for at least 7, 14, 21 or 49 days). Abbreviations: bOPV, bivalent oral poliovirus vaccine; IPV, inactivated poliovirus vaccine; PV2, polyvirus type 2; tOPV, trivalent oral poliovirus vaccine.

There were no children excreting PV2 during baseline stool collection (prior to tOPV administration). Excretion of PV2 in stool and its duration is shown in Figure 2. The excretion was highest on day 7 (75, 68, and 73% for study arms A, B, and C, respectively); excretion decreased with every subsequent stool sampling and was 7–10% on day 49. The mean duration of PV2 excretion in days was 11.0 (95% CI, 8.9–13.1), 10.5 (95% CI, 8.4–12.6), and 11.2 (95% CI, 9.0–13.4) for study arms A, B and C, respectively. There were no statistical differences between the study arms either in the proportion of children excreting PV2 nor in the duration of PV2 excretion. Children from arm B who had excreted PV1 or PV3 on the day of the tOPV challenge had a higher probability of PV2 excretion at any time following the tOPV challenge than those children from arm B who had not excreted PV on the day of the tOPV challenge (58/74, [78%] vs. 29/42 [62%], *P* = .2) and had a longer duration of PV2 excretion (12.4 days, 95% CI, 5.8–19 vs. 6.6 days, 95% CI, 1.6–11.6; *P* = .008). While the difference in the duration of excretion reached statistical significance, the difference in the overall rate of excretion did not.

**Figure 3.**
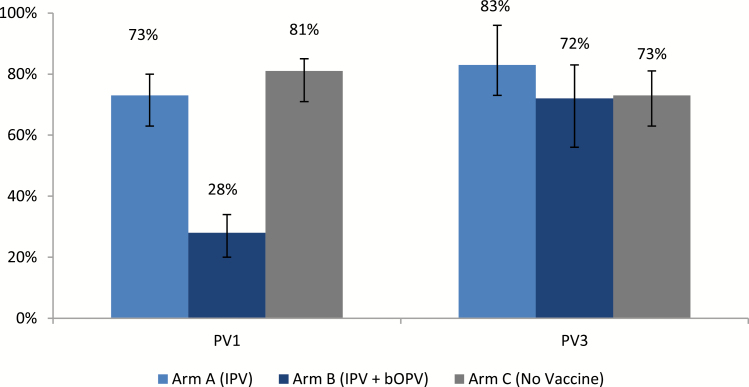
Excretion of PV1 and PV3 following challenge with tOPV (at any point after the tOPV challenge). Abbreviations: bOPV, bivalent oral poliovirus vaccine; IPV, inactivated poliovirus vaccine; PV1, polyvirus type 2; PV3, polyvirus type 3; tOPV, trivalent oral poliovirus vaccine.

For the analysis of excretion of PV1 and PV3, we excluded children who were shedding a type-specific poliovirus prior to tOPV administration (excluded were 1/113 from study arm A, 66/114 from study arm B, and 1/103 from arm C; the high proportion of excluded children in arm B is expected, because these were bOPV recipients). We present an analysis of the excretion of PV1 or PV3 at any point following the tOPV challenge (Figure 3). Children in study arm B excreted PV1 significantly less often than children in the other 2 arms (*P* <0.001); this significance, however, was absent for PV3 (*P* = .5).

## DISCUSSION

In our study, we did not observe a reduction in the excretion of PV2 after the tOPV challenge in children who had received IPV with bOPV, as compared to those who had received IPV alone or had not received any prior poliovirus vaccine. There was no observed cross-mucosal immunity for PV2 when a combination of IPV and bOPV was administered.

We observed excellent seroconversion achieved after either 1 IPV dose or with 1 combination dose of IPV and bOPV. Especially, seroconversion for PV2 after 1 dose of IPV was higher than previously reported in Cuba (95% in our study compared with 63% reported earlier) [21]. This difference is likely attributed to the higher age (6 months) of the first IPV dose, when interference with maternal antibodies is already minimal as compared to the age of 4 months in the previous study [22].

Study arm C had unexpectedly high serological response to a single tOPV dose (>95% for all serotypes). This was described earlier in Cuba; because tOPV is administered in mass campaigns in Cuba, its immunogenicity is increased [23]. It is also likely that the hygiene and social-economic situation in Cuba has improved further, supporting better serological responses to OPVs [24–26].

As expected, the excretion of PV1 after the tOPV challenge was significantly reduced in children who had received bOPV previously; this, however, was not the case for PV3, perhaps because PV1 is a more dominant serotype than PV3 and, at first vaccination contact, it induced an intestinal response more readily than PV3 [27].

Our study had some limitations. Due to later-than-expected completion of recruitments, the study procedures were still ongoing when the first NID started in Camaguey; therefore, it is possible that some of the recruited children were exposed to the vaccine polioviruses from the environment.

A polio eradication program cannot assume PV2 mucosal immunity with the current polio immunization schedule. Outbreaks involving cVDPV2 need to be responded to with a monovalent type 2 OPV vaccine in order to interrupt cVDPV2 transmission. Our study demonstrated excellent humoral immune responses to IPV, either alone or in combination with bOPV, providing further evidence that the new routine immunization schedule based on the polio endgame strategy [7] is sufficiently protective against paralysis caused by types 1 or 3 of wild or vaccine-derived polioviruses.

## Notes

***Acknowledgments.*** The authors thank the Government of Cuba, its Ministry of Health, Health Authorities of Camaguey Province, and the Cuban office of the Pan American Health Organization for their support of this study. They thank the external monitor of the study, Laureano Mestra, who contributed significantly to the success of the trial, and the parents and infants who participated in the trial.

***Disclaimer.*** This study was approved by the Ethics Review Committee of the World Health Organization in Geneva, by the Internal Review Board of the Pedro Kouri Institute in Havana, and by the Provincial Center for Hygiene and Epidemiology, Camaguey.

***Financial support.*** This work was supported by the World Health Organization, Geneva and the Government of Japan.

***Supplement sponsorship.*** This article appears as part of the supplement “Polio endgame and beyond: Vaccine choices, transmission dynamics, and surveillance implications,” sponsored by The Bill & Melinda Gates Foundation.

***Potential conflicts of interest.*** All authors: No reported conflicts of interest. All authors have submitted the ICMJE Form for Disclosure of Potential Conflicts of Interest. Conflicts that the editors consider relevant to the content of the manuscript have been disclosed.
